# A new species of *Petrolisthes* (Crustacea, Anomura, Porcellanidae) inhabiting vermetid formations (Mollusca, Gastropoda, Vermetidae) in the southern Caribbean Sea

**DOI:** 10.3897/zookeys.876.37244

**Published:** 2019-09-25

**Authors:** Alexandra Hiller, Bernd Werding

**Affiliations:** 1 Smithsonian Tropical Research Institute, Apartado 0843-03092, Panamá, República de Panamá Smithsonian Tropical Research Institute Panama Panama; 2 Institut für Tierökologie und Spezielle Zoologie der Justus-Liebig-Universität Giessen, Heinrich-Buff-Ring 29 (Tierhaus), D-35392 Giessen, Germany Justus-Liebig-Universität Giessen Germany

**Keywords:** West Atlantic, *Petrolisthes virgilius* sp. nov., *Petrolisthes tonsorius*, ecological differences, color morphs, vermetid formations, mitochondrial marker

## Abstract

*Petrolisthes virgilius***sp. nov.** from the Caribbean Sea of Colombia is described. The new species resembles *P. tonsorius* morphologically but differs from it principally by its color and habitat. *Petrolisthes tonsorius* is brown or blueish brown and occurs under intertidal boulders strongly exposed to water movement. *Petrolisthes virgilius***sp. nov.** is pale brown to beige and lives exclusively in intertidal areas dominated by vermetid snails, exposed to heavy wave action. The entangled tubular shells of vermetids are cemented to each other and to a hard substrate like beach rock, forming a microhabitat for the new crab species and other porcellanids of the genera *Neopisosoma* and *Clastotoechus*. Large genetic distances between DNA sequences of the mitochondrial 16S rDNA gene from *P. virgilius***sp. nov.** and *P. tonsorius* confirmed that they comprise different species. *Petrolisthes virgilius***sp. nov.** is the 53^rd^ member of the West Atlantic porcellanid fauna.

## Introduction

**Figure 1.**
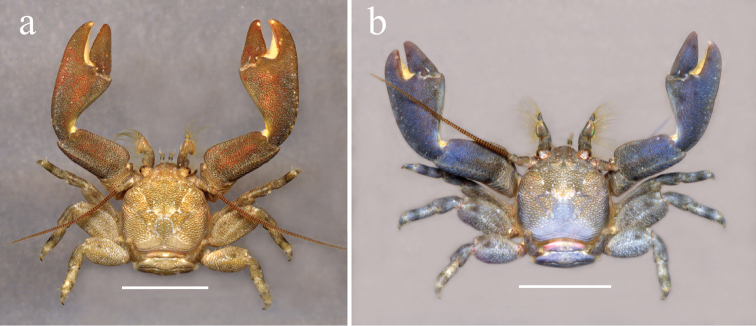
*Petrolisthes tonsorius*, Venezuela, Isla Cubagua. **a** Brownish color morph **b** Blueish color morph. Scale bars: 3.7 mm (**a**); 3.2 mm (**b**).

**Figure 2.**
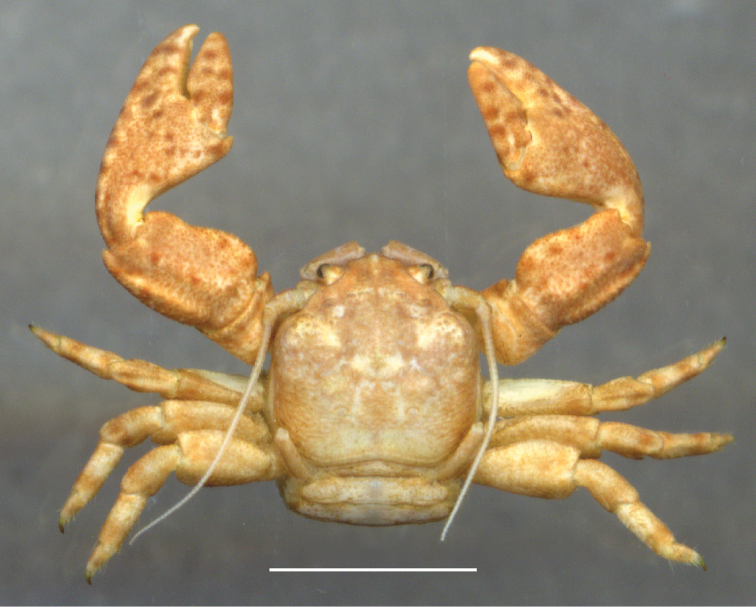
*Petrolisthes virgilius* sp. nov., Colombia, Santa Marta. Scale bar: 2.4 mm.

The porcellanid fauna of the western Atlantic has been studied intensively in the last 60 years. With the last additions by Ferreira and Tavares (2017, 2019) and Werding and Hiller (2017), consisting of three new species of *Pachycheles* Stimpson, the number of western Atlantic species rose to 52. Werding (1978) found individuals of *Petrolisthes* inhabiting vermetid formations in the Colombian Gulf of Urabá, and assigned them to *P. tonsorius* Haig, 1960, but warned that coloration and habitat of the crab specimens were atypical. *Petrolisthes tonsorius* inhabits the rocky intertidal of both the Caribbean and the tropical East Pacific and exhibits a brown to dark-brown color (Fig. 1a), sometimes blueish (Fig. 1b). The individuals found inhabiting vermetid formations were pale brown to beige (Fig. 2). Morphological re-examination of this color morph, and genetic distances estimated between DNA sequences of the 16S rDNA gene from this new form and *P. tonsorius* confirmed that they comprise different species. Here we describe the new form as *Petrolisthes virgilius* sp. nov.

## Materials and methods

Material of *Petrolisthes virgilius* sp. nov. collected in the Colombian regions of Santa Marta and the Gulf of Urabá was used for morphological examination and molecular analyses. Type material was deposited in the collection of the Museo de Historia Natural Marina de Colombia (INV CRU), INVEMAR (Institute of Marine and Coastal Research of Colombia, Santa Marta). Specimens were sexed and measured by using a stereoscope with a micrometer. Measurements are given in mm and correspond to carapace length, followed by carapace width.

DNA was extracted from the chelipeds or walking legs of seven specimens of the new species (3 from Santa Marta and 4 from the Gulf of Urabá) using the DNeasy Blood & Tissue Kit (Qiagen), following the manufacturer´s protocol for animal tissues. A 540 bp fragment of the ribosomal 16S rDNA was amplified using primers 16Sar (CGCCTGTTTATCAAAAACAT) and 16Sbr (CCGGTCTGAACTCAGATCACGT) (Palumbi 1996), and trimmed to 496 bp. Double-stranded amplifications were performed in 12 ml volume reactions containing 2.5 µl of Taq buffer (5×), 1.7 µl of dNTP mix (8 mM), 0.6 µl of each primer (10µM), 1.2 µl of MgCl_2_ (25mM), 0.2 µl of GoTaq Flexi DNA *Taq* Polymerase (Promega), 1 µl of DNA template, and 4.8 µl of ddH_2_0. Thermal cycling conditions consisted of an initial denaturation step at 96 °C for 3 min, followed by 30 cycles of 95 °C for 1 min, 50 °C for 1 min, and 72 °C for 1 min. An extension step at 72 °C for 5 min followed the last cycle. PCR products were cleaned using the ExoSap-IT kit (USB Corporation) following the manufacturer´s protocol. Clean PCR products were cycle-sequenced in both directions using the BigDye Terminator v. 3.1 Cycle Sequencing Kit, and electrophoresed in an Applied Biosystems 3130 Genetic Analyzer.

The 16S rDNA sequences of *P. virgilius* sp. nov. were compared to two sequences of *P. tonsorius* from the Venezuelan Caribbean and the Colombian Pacific, published by Hiller et al. (2006) (GenBank accession numbers DQ444960 and DQ444959). The BioEdit Sequence Alignment Editor (Hall 1999) was used to trim primer regions from DNA sequences. MAFFT v. 7 (Katoh and Standley 2013) was used to align the sequences of *P. virgilius* sp. nov. and *P. tonsorius*. Genetic distances within and between these species were estimated using Kimura’s two-parameter model (K2P; Kimura 1980) implemented in MEGA v. 7.0 (Kumar et al. 2016). GenBank accession numbers of the 16S rDNA sequences of *P. virgilius* sp. nov. are MN275526-275532.

## Systematics

### Family Porcellanidae Haworth, 1825

#### Petrolisthes virgilius sp. nov.

Taxon classificationAnimaliaDecapodaPorcellanidae

6DA9AD71-98C0-5645-841D-49A79572A776

http://zoobank.orgE0018D07-579A-4A53-84D1-2EF333D59098

Figs 2 , 3a–e

Petrolisthes tonsorius Werding, 1978: 220 (not P. tonsorius Haig, 1960: 85–88)

##### Material.

**Holotype**: male, INV CRU8404, Colombia, Chocó, Gulf of Urabá, Triganá, Napú, coll. J. Lazarus, 05 Dec. 2010; 4.6 × 4.5 mm.

**Figure 3.**
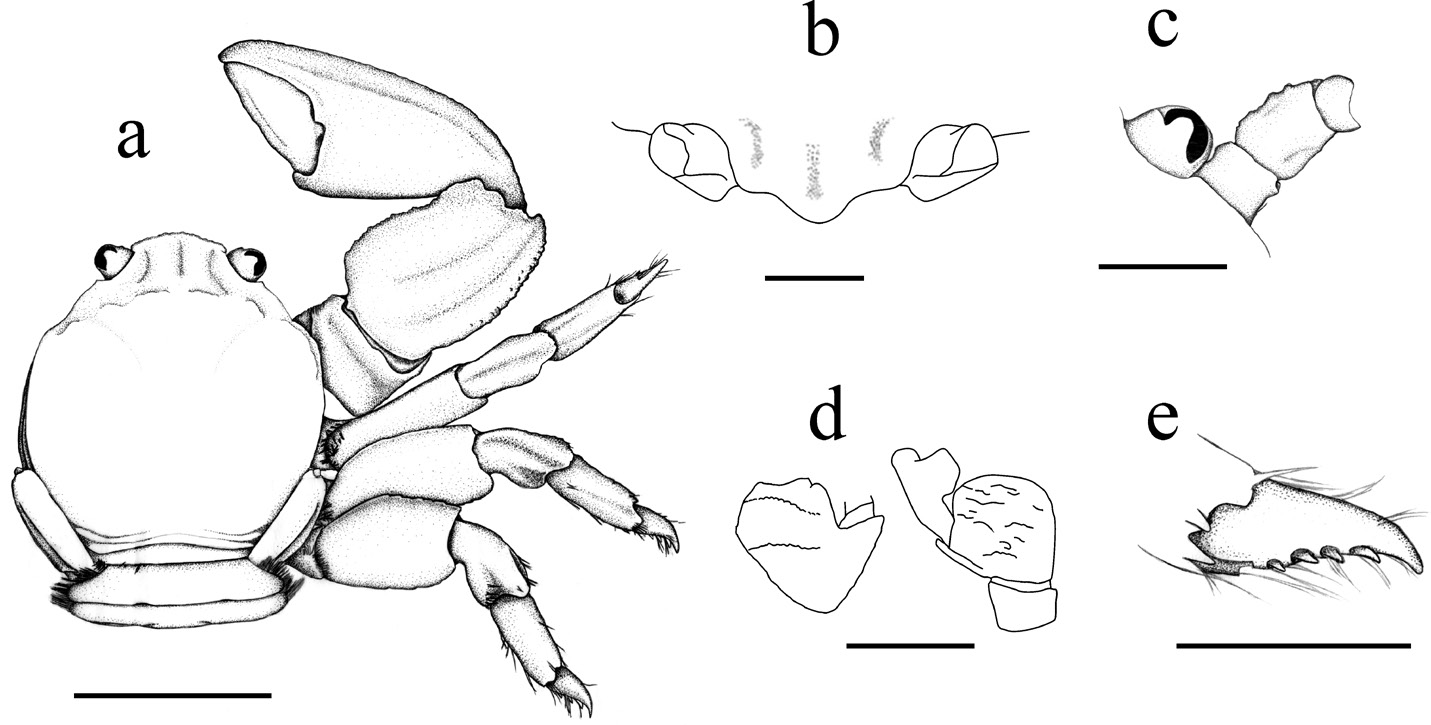
*Petrolisthes virgilius* sp. nov., female (ovigerous) paratype, INV-CRU 8405, Colombia, Chocó, Gulf of Urabá, Triganá, Napú. **a** Dorsal view **b** rostrum, frontal view **c** orbit with basal segments of antenna **d** basal segment of antennular peduncle **e** dactylus of last right walking leg. Scale bars: 5.0 mm (**a**); 1.0 mm (**b–e**).

**Paratypes**: 2 males, 2 females (1 ovigerous), INV CRU8405, same collection data as holotype. Size of males is 4.2 × 3.7 mm and 3.3 × 3.2 mm; size of females is 5.3 × 5.2 mm (ovigerous) and 4.3 × 4.0 mm (Fig. 3).

Sizes of largest male and female reported by Werding (1978) are, respectively, 8.0 × 8.1 mm and 6.5 × 6.1 mm.

##### Diagnosis.

Carapace subquadrate, its margins subparallel posterior to epibranchial angle, nearly smooth, covered anteriorly with few flattened granules; no epibranchial spine; front narrow, triangular, with deep median groove; carpus 1½ times as long as wide, surface granulate, anterior margin with a broad, rounded lobe, separated through an indentation from a shallow distal lobe; manus with a longitudinal ridge; fingers blunt, outer margin convex, forming a rounded crest along entire length; merus of walking legs unarmed.

##### Description.

Carapace about as long as broad, subquadrate, lateral margins subparallel posterior to epibranchial angles; nearly smooth, covered anteriorly with few flattened granules and posteriorly with light plications; grooves marking the various regions distinct. No epibranchial spine. Front narrow, triangular, strongly produced, with a deep median groove extending between protogastric lobes; no supraocular spine; inner orbital angle not produced. Orbits rather shallow; outer orbital angle produced into a small tooth. Eyes moderately large. Carapace naked. Basal segments of antennae thick, granulate, first movable segments with a marked crest produced to distal edge, second massive and cylindrical, flagellum about 1½ as long as carapace.

Chelipeds broad, naked, covered with small flattened granules on dorsal surface, smooth ventrally. Merus with a small granular lobe on anterior margin, inner distal edge not produced. Carpus ca. 1½ times as long as broad, surface granulate, anterior margin produced into a broad, rounded lobe extending 2/3 of its length, and separated through an indentation from a shallow distal lobe; dorsal surface with a broad, longitudinal ridge. Posterior border convex, forming a granulate crest, ending distally in a rounded tooth. Chelae subequal, moderately large. Manus with a longitudinal ridge. Fingers blunt, pollex frequently longer than dactylus, outer margin convex, forming a rounded crest along entire length; dactylus longitudinally notched. Gape without pubescence.

Walking legs compact, with scattered simple setae. Merus unarmed, broad, flattened; carpus and propodus naked, crested above, dactylus with four movable spinules on inner border.

Telson with seven plates.

##### Coloration.

The overall coloration of *P. virgilius* sp. nov. is pale brown to beige, the brown coloration prevailing on chelipeds and frontal half of carapace (Fig. 2). Carpus and manus of chelipeds frequently show scattered, dark-brown spots.

##### Ecology.

*Petrolisthes virgilius* sp. nov. was found exclusively in intertidal formations of vermetid snails exposed to strong waves. The new species shares this habitat with *Neopisosoma angustifrons* (Benedict, 1901), *N. neglectum* Werding, 1986, and *Clastotoechus nodosus* (Streets, 1872). The latter species can also be found in other intertidal fouling incrustations in heavily wave-exposed rocky shores (Werding 1986).

##### Distribution.

Colombia, Santa Marta and Gulf of Urabá regions.

##### Etymology.

The new species is named after Dr. Virgilio Galvis Ramírez MD for his support and interest in our research on marine crabs, and for his contributions to medical sciences in Colombia.

##### Molecular analysis.

The 496 bp alignment consisting of seven 16S rDNA sequences of *Petrolisthes virgilius* and two of *P. tonsorius* revealed two haplotypes within the new species, which differ only by one nucleotide. One haplotype was more frequent than the other and was shared by two individuals from Santa Marta and three from the Gulf of Urabá. The other haplotype was shared by two individuals from Santa Marta. The average of genetic distance within the new species was 0.2%. Distances between *P. virgilius* and *P. tonsorius* ranged between 9.6% and 10.2%. The average distance between populations of *P. tonsorius* from the Pacific and Caribbean was 4.8%.

##### Remarks.

*Petrolisthes virgilius* can be distinguished morphologically from *P. tonsorius* and similar species by the marked granulation of carapace and extremities, the marked indentation of the anterior margin of the cheliped’s carpus, and the accentuated crests of the chelipeds.

## Discussion

The new species belongs to one of the morphological lines in the diverse and world-wide distributed genus *Petrolisthes*. This morphological line is strictly American and includes species lacking spines and dentations in carapace and extremities. In the West Atlantic this group is represented by two trans-isthmian species, *P. tridentatus* Stimpson, 1859, and *P. tonsorius* Haig, 1960, and by *P. quadratus* Benedict, 1901, *P. gertrudae* Werding, 1996, *P. hispaniolensis* Werding & Hiller, 2005, and now *P. virgilius*. Besides the two trans-isthmian species, the East Pacific members of this group are numerous, and are morphologically represented by the tropical *P. galapagensis* Haig, 1960, and a number of subtropical, warm-temperate and temperate species (see Haig 1960). Despite the close resemblance between *P. virgilius* and *P. tonsorius*, which led Werding (1978) to cautiously consider them conspecific, the large 16S genetic distance and distinguishing color and habitat confirms that they are different species. The genetic distance is double to that between individuals of *P. tonsorius* from each side of the Isthmus of Panama and surpasses by far the distance of 1.5% found by Hiller et al. (2006) for the trans-isthmian *P. armatus* Gibbes, 1850. The high molecular divergence between *P. virgilius* and *P. tonsorius* seems to be accompanied by species-specific coloration. However, intraspecific variation in color in *P. tonsorius* from each side of the Isthmus of Panama overlaps and therefore, in this case, color does not distinguish populations from each ocean. The crabs from both the East Pacific and West Atlantic display carapace and extremities that vary from brown to blueish. Haig (1960) described *P. tonsorius* from preserved specimens collected in the Galápagos Islands and surmised that they were blueish-colored in life. She wrote (p. 87): “After more than twenty years in alcohol, a few specimens show a dark blue-violet on the metabranchial regions of the carapace, ringed by a darker line of the same color; this shade of blue is also present on the eyestalks, movable segments of the antennae, walking legs, telson of the abdomen, and palps of the maxillipeds”. Color and color pattern have been taxonomically reliable characters for distinguishing cryptic species of porcellanids (Hiller et al. 2006; Hiller and Werding 2007) and other anomurans like hermit crabs (Malay et el. 2012; Negri et al. 2014) and squat lobsters (Macpherson and Machordom 2001; Cabezas et al. 2011). However, in some species complexes in Anomura color may not vary interspecifically (Werding and Hiller 2017) or it can vary intraspecifically (Rodríguez-Flores et al. 2018).

*Petrolisthes virgilius* is ecologically unique compared to its morphological allies, typically occurring under intertidal boulders moderately to highly exposed to water movement. The only other species in association with living organisms is *P. gertrudae*, occasionally found on *Zoanthus sociatus* (Ellis, 1768) (see Werding 1996). The vermetid conglomerates where *P. virgilius* was found in Santa Marta and the Gulf of Urabá are cemented to a beachrock platform exposed to strong waves, which provide the vermetids and associated fauna with oxygen and nutrients. This singular vermetid habitat provides shelter to other porcellanid species of genera *Neopisosoma* Haig, 1960, and *Clastotoechus* Haig, 1960. The new species seems to have evolved in tight association to intertidal vermetid formations exposed to extremely strong water movement.

Including the new species, the genus *Petrolisthes* now comprises 111 species and the family Porcellanidae 305 species.

## Supplementary Material

XML Treatment for Petrolisthes virgilius
